# Cancer survivors not participating in observational patient-reported outcome studies have a lower survival compared to participants: the population-based PROFILES registry

**DOI:** 10.1007/s11136-018-1979-0

**Published:** 2018-08-30

**Authors:** Belle H. de Rooij, Nicole P. M. Ezendam, Floortje Mols, Pauline A. J. Vissers, Melissa S. Y. Thong, Carla C. P. Vlooswijk, Simone Oerlemans, Olga Husson, Nicole J. E. Horevoorts, Lonneke V. van de Poll-Franse

**Affiliations:** 1 Department of Medical and Clinical Psychology, CoRPS - Center of Research on Psychology in Somatic Diseases, Tilburg University, Warandelaan 2, PO Box 90153, 5000 LE Tilburg, The Netherlands; 2 The Netherlands Comprehensive Cancer Organisation, Utrecht, The Netherlands; 3 Department of Medical Psychology, Academic Medical Center University of Amsterdam, Amsterdam Public Health Research Institute, Amsterdam, The Netherlands; 4 Department of Medical Psychology, Radboud University Medical Center, Nijmegen, The Netherlands; 5 Division of Psychosocial Research and Epidemiology, The Netherlands Cancer Institute, Amsterdam, The Netherlands

**Keywords:** Patient-reported outcomes, Cancer survivors, Non-participation, Non-response bias, Quality of life

## Abstract

**Purpose:**

The ‘Patient Reported Outcomes Following Initial treatment and Long-term Evaluation of Survivorship’ (PROFILES) registry collects patient-reported outcomes (PROs) from short- and long-term cancer survivors in the Netherlands, in a population-based setting. The aim of this analysis is to assess the generalizability of observational PRO research among cancer survivors by comparing socio-demographic and clinical characteristics, and survival of participants and non-participants in cancer survivors invited for questionnaire research through the PROFILES registry.

**Methods:**

Between 2008 and 2015, cancer survivors with different cancer diagnoses (*N* = 14,011) were invited to participate in PROFILES registry studies, of whom 69% (*N* = 9684) participated. Socio-demographic and clinical characteristics and survival data, collected through the Netherlands Cancer Registry, were associated with participation versus non-participation in multivariable logistic regression analyses and cox proportional hazard regression models, respectively.

**Results:**

Participants had a significantly better survival compared to non-participants (HR = 1.47, *P* < .01). Participation was associated with male gender, being 60–70 years old, high socio-economic status, receiving any treatment, receiving radiotherapy, having no comorbidities, and a cancer diagnosis 2–3 years before invitation. Sensitivity analysis demonstrates that the health-related quality of life (HRQoL) might be up to 1.3 points lower (scale 0–100) using hot deck imputation compared to non-imputed participant data.

**Conclusions:**

Cancer survivors not participating in observational PROs research significantly differ from participants, with respect to socio-demographic and clinical characteristics, and survival. Their HRQoL scores may be systematically lower compared to participants. Therefore, even in PRO studies with relatively high participation rates, observed outcomes may represent the healthier patient with better outcomes.

## Introduction

Patient-reported outcomes (PROs) are increasingly being used in patient-centered outcome research to support informed health care decisions [1]. Relying on individual patient participation, PROs are subject to bias, of which non-response bias has raised high concerns [2]. If certain patients are underrepresented in PRO research, the generalizability of the outcomes are likely to be affected, which may in turn negatively impact the usability in (shared) informed decision making [3, 4]. Yet, few attempts have been made to quantify and interpret associations with non-participation in PRO research [5, 6].

The ‘Patient Reported Outcomes Following Initial treatment and Long-term Evaluation of Survivorship’ (PROFILES ) registry is a large and dynamic population-based cohort for the study of the physical and psychosocial impact of cancer and its treatment [7]. Since 2008, the PROFILES registry has been used to collect PROs among both short- and long-term cancer survivors in observational population-based studies in the Netherlands. Over 20,000 individuals having cancer at 16 different cancer sites were approached to date. Complete and comprehensive supplemental data on socio-demographics, clinical characteristics, and survival are available for the full population of participants, as well as for the non-participants, through the Netherlands Cancer Registry (NCR) and linkage with the Dutch municipal personal records database.

Although it has been widely acknowledged that selection bias is present in observational PRO research, few studies among cancer survivors have access to non-participant data to assess whether their sample was representative and use strategies to adjust for non-participation bias [8–12]. Furthermore, beyond basic socio-demographic data and sometimes limited clinical data, little is known about health-related quality of life (HRQoL) and survival of participants and non-participants in observational PRO research among cancer survivors. Information about (long-term) survival could provide new insights into the potentially unmeasured differences in HRQoL between participants and non-participants at time of invitation for a questionnaire. Based on the notion that individuals with a poorer health status may be less likely to participate in studies [2–4], it was hypothesized that non-participants have a lower survival than participants that cannot be explained by differences in cancer stage or treatment at diagnosis alone. If true, cancer survivors with low survival and potentially poor initial HRQoL are underrepresented in observational PRO research.

The aim of the current study is to investigate characteristics and survival of participants compared to non-participants, among both short- and long-term cancer survivors invited for questionnaire research through the population-based PROFILES registry.

## Methods

### Design/setting

Data from the PROFILES registry were used. PROs are collected within a sampling frame of the NCR and can be linked with clinical data of all individuals newly diagnosed with cancer in the Netherlands [13]. The PROFILES registry started data collection of the first cohort of cancer survivors in 2008 and is still ongoing, including studies on various cancer types.

### Data collection

A detailed description of the data collection has been described previously [7]. In brief, in each study sample, cancer survivors were informed about the study via a letter by their (ex-)attending specialist. This letter contained either an informed consent form and a paper questionnaire, or a secured link to a web-based informed consent form and online questionnaire. In study samples where the secured link was provided, patients could return a postcard to request a paper-and-pencil questionnaire. Data from the PROFILES registry are freely available for non-commercial scientific research, subject to study question, privacy and confidentiality restrictions, and registration (http://www.profilesregistry.nl).

### Study population

The current analyses include 12 study samples from the PROFILES registry in which similar core PRO questionnaires and methodology of data collection were used, with inclusion between May 2008 and April 2015. Table 1 describes the number of cancer survivors, inclusion criteria, cancer type, research purpose, design, and questionnaires collected by study sample (Table 1). In all study samples, participants were excluded if they were not able to complete a Dutch questionnaire according to their current or former (if not under follow-up) attending specialist (i.e., cognitive impairment, non-native speaker, too ill to participate). Individuals that died or emigrated prior to the start of the study were excluded, according to data from the hospital of diagnosis and/or data from the Dutch municipal personal records database. The Dutch municipal records database collects mortality and residential data from all citizens through municipal registries. Further, some patients (*N* = 10) could not be linked to clinical data from the NCR and were therefore excluded from analysis. If the same individual was invited for participation in multiple studies, data of the first questionnaire were included in the current analysis. Ethical approval was obtained for all study samples separately, from a local certified medical ethics committee.

**Table 1:** Study sample characteristics

	Cancer type(s)	Inclusion criteria	Inclusion date	Time since diagnosis, years, *M* (SD)	Total, *N*	Participants, *N*	Non-participants^a^		Participation rate of total (%)	Participation rate of those verifiable^b^ (%)
							Verifiable, *N*	Unverifiable, *N*		
1	Endometrial cancer	Diagnosed between 1998 and 2007 Stage I or II	From May 2008 to May 2009	4.7 (2.4)	1083	739	222	122	68	77
2	Colon cancer/rectum cancer	Diagnosed between 1998 and 2007 Weighted random sample based on cancer type and sex Oversampling of short time since diagnosis	From January 2009 to June 2009	4.1 (2.5)	1807	1363	302	142	75	82
3	Hodgkin lymphoma/non-Hodgkin lymphoma/Chronic lymphocytic leukemia/multiple myeloma	Diagnosed between 1999 and 2008 18–85 years	From May 2009 to August 2009 and from November 2009 to February 2010	4.1 (2.8)	1711	1142	333	236	67	77
4	Colon cancer/rectum cancer	Diagnosed between 2000 and 2009	November 2010	5.2 (2.8)	3528	2593	610	325	73	81
5	Thyroid cancer	Diagnosed between 1990 and 2008	From November 2010 to February 2011	9.7 (5.5)	456	301	48	107	66	86
6	Endometrial cancer/Ovarian cancer	Newly diagnosed Non-palliative	From April 2011 to March 2014	0.3 (0.8)	533	389	144	0	73	73
7	Hodgkin lymphoma/non-Hodgkin lymphoma/Chronic lymphocytic leukemia/multiple myeloma	Diagnosed between 2008 and 2013 18–85 years	May 2011, from May 2012 to August 2012, and May 2013	1.8 (0.7)	862	608	165	89	71	79
8	Prostate cancer	Diagnosed between 2006 and 2009 Stage cT1–cT3	From October 2011 to February 2012	4.0 (1.2)	997	691	255	51	69	73
9	Ovarian cancer/Borderline ovarian cancer	Diagnosed between 2000 and 2010	From February 2012 to April 2012	6.4 (3.1)	530	263	267	Unknown	50	Unknown
10	Diffuse large B-cell lymphoma	Diagnosed between January 2007 and July 2012 Age > 18 years	December 2012	3.0 (1.6)	298	145	153	Unknown	49	Unknown
11	basal cell/squamous cell cancer	Diagnosed between 2013 and 2014 80% basal cell, 20% squamous cell cancer 80% facial skin lesion, 20% non-facial skin lesion	June 2014	1.0 (0.5)	1157	715	258	184	62	73
12	Prostate cancer/ melanoma	Diagnosed between 2005 and 2014 No diagnosis of prostate cancer during surgery for bladder cancer (incidental finding) 18–85 years All stages	From November 2014 to April 2015	4.1 (1.5)	1049	735	314	Unknown	70	Unknown
Total				4.1 (3.0)	14,011	9684	3071	1256	69	76

^a^Non-participants were unverifiable when registered address did not correspond with national zip code registration. For three study samples, no address check had been done (unverifiable unknown)

^b^Participation rate of those verifiable is calculated by dividing all participants by the participants and verifiable non-participants

### Measures

Patients invited for participation, the questionnaires received, and patients for whom the address was unverifiable were collected through the PROFILES registry. Address checks had been done to verify whether the registered address corresponded with national zip code registration. In three study samples unverifiable addresses have not been determined (Table 1). Participants included all individuals that returned the questionnaire. Non-participants included all individuals that did not return the questionnaire, including those for whom the address was unverifiable. Initial date of invitation for questionnaire participation was registered for all (non-)participants in PROFILES.

Socio-demographic and clinical data were obtained from the NCR, while mortality data were obtained from the Dutch municipal personal records database. Socio-demographic variables include date of birth, sex, and socio-economic status (SES). SES was based on postal code of the residence area of the patient, combining aggregated individual fiscal data on the economic value of the home and household incomes, and was categorized into low, medium, high, or institutionalized/unknown [14].

Clinical data include tumor type, stage, primary treatments received, date of diagnosis, and comorbidities at time of diagnosis. Tumor type was classified according to the third International Classification of Diseases for Oncology (ICDO-3) [15], and cancer stage was classified according to TNM [16] or Ann Arbor Code (Hodgkin lymphoma and Non-Hodgkin lymphoma). TNM 5 was used for patients diagnosed from 2002 to 2003, TNM 6 for patients diagnosed from 2003 to 2010, and TNM 7 was used for patients diagnosed from 2010. For Chronic Lymphocytic Leukemia, Multiple Myeloma, and borderline ovarian cancer, stage was not determined nor registered. Primary treatments received were classified into surgery, systemic therapy (chemotherapy, targeted therapy, immune therapy), radiation therapy (including brachytherapy), hormone therapy, no treatment/active surveillance or unknown. Comorbidity was classified using a modified version of the Charlson Index [17] and categorized into no comorbidity, 1 comorbidity, or more than 1 comorbidity. Patients being alive at time of analysis, patients that died during follow-up, and date of death were obtained from the Dutch municipal personal records database and was last verified on February 1, 2017.

Age at time of questionnaire invitation was determined by the difference in patients’ date of birth (obtained from NCR) and date of invitation for questionnaire participation. The time since diagnosis at time of questionnaire invitation was categorized into four quartiles: 0–2 years, 2–3 years, 3–5 years, and > 5 years.

The EORTC QLQ-C30 (version 3.0) was used to assess health-related quality of life (HRQoL) in the participants [18]. The scores were linearly transformed into a score between 0 and 100 [19].

### Statistical analyses

Statistical analyses were conducted using SAS version 9.4. (SAS Institute, Cary, NC, 1999). For the baseline characteristics, frequencies with percentages and means with standard deviations were used to describe the variables, and Chi-square tests and independent t-tests were used to test the differences between participants and non-participants.

Socio-demographic (age, sex, SES) and clinical (cancer type, stage, primary treatments received, time since diagnosis, number of comorbidities, mortality) characteristics associated with participation versus non-participation were assessed in a multivariable logistic regression model (including patients with unverifiable addresses).

Graphs were used to present response rates according to age at invitation or time since diagnosis, stratified for sex, SES, comorbidities, and cancer stage. In order to capture patterns in the data, the graphs were smoothened by calculating the central moving mean for each age by averaging the participation rates of the 5 previous and the 5 upcoming values.

Kaplan–Meier curves of the unadjusted survival function were estimated for participants and non-participants. Cox proportional hazard regression models were conducted to assess the unadjusted and adjusted differences in all-cause mortality between participants and non-participants. Survival duration was specified as time from invitation for participation in a study until either death or censoring date (February 1, 2017). Because time between diagnosis and invitation for participation in the study was highly variable (0–12 years), patients with a shorter time since diagnosis might have a higher mortality risk compared to patients that already lived longer after diagnosis [20]. To adjust for this survivorship bias, a variable with the left-truncation time (time between diagnosis and invitation for participation in the study) was added as an argument and time of invitation for participation was set as entry time [21]. The model was additionally adjusted for covariates controlling for factors influencing survival. In addition, subgroup analyses were conducted to assess whether survival between participants and non-participants differed between age groups.

In addition, the differences in HRQoL of participants versus non-participants were estimated. As HRQoL of non-participants were naturally not available, their scores were estimated by matching with participants using a ‘hot deck’ approach. An advantage of the ‘hot deck’ approach is that it uses real observed data from similar individuals and, unlike other imputation techniques, avoids strong parametric assumptions [22]. Participants who completed the EORTC QLQ-C30 were matched to non-participants (on age [5-year strata], survival [2-year strata], sex and cancer type; matching ratio 1:1, randomly selected). A sensitivity analysis compared the HRQoL of participant data with imputed data (participants + matched non-participants).

## Results

In total, 14,011 cancer survivors were invited for participation in one of 12 cohort studies of the PROFILES registry. Overall, 69% (*N* = 9684) of the total population completed the questionnaire, but participation rates of individual study samples varied between 49 and 75%. When patients were excluded with unverifiable addresses, the overall participation rate was 76% (Table 1).

In multivariable logistic regression analysis, younger and older patients were less likely to participate than patients aged 60–70 years, men more often participated than women and patients with a medium or high SES were more likely to participate than patients with a low SES. Further, patients with (borderline) ovarian cancer were less likely to participate compared to colon cancer patients. Patients treated with radiotherapy were more likely to participate, whereas patient who had received no treatment were less likely to participate. Patients were more likely to participate when they were 2–3 after diagnosis compared to < 2 years or > 3 years after diagnosis. Finally, patients with 2 or more comorbid conditions were less likely to participate (Table 2).

**Table 2:** Odds ratios (OR) of participants versus total non-participants, multivariable logistic regression

	Participants *N* = 9684	Non-participants *N* = 4327	Odds of participation versus non-participation	
	*N* (%)	*N* (%)	OR	95% CI
Age at invitation				
< 50 years	761 (8)	571 (13)	0.47**	0.42–0.56
50–60	1372 (14)	573 (13)	0.85**	0.75–0.96
60–70	3136 (32)	1086 (25)	1.00 (ref)	
70–80	3306 (34)	1345 (31)	0.87**	0.79–0.96
> 80	1109 (11)	752 (17)	0.58**	0.51–0.65
Sex				
Male	5076 (52)	1991 (46)	1.00 (ref)	
Female	4608 (48)	2336 (54)	0.82**	0.75–0.89
SES				
Low	1900 (20)	1073 (25)	1.00 (ref)	
Medium	3765 (39)	1691 (39)	1.21**	1.10–1.33
High	3436 (35)	1185 (27)	1.53**	1.38–1.70
Unknown/institutionalized	585 (6)	379 (9)	1.98	0.84–1.15
Cancer type				
Colon	2483 (26)	917 (21)	1.00 (ref)	
Rectum	1470 (15)	460 (11)	0.98	0.84–1.14
Melanoma	244 (3)	120 (3)	0.81	0.63–1.04
Basal/squamous cell	715 (7)	442 (10)	1.36	1.00–1.86
Endometrial	956 (10)	416 (10)	0.88	0.75–1.04
Ovarian	353 (4)	232 (5)	0.63**	0.51–0.77
Ovarian borderline	82 (1)	105 (2)	0.39**	0.27–0.57
Prostate	1182 (12)	500 (12)	0.88	0.71–1.08
Thyroid	303 (3)	155 (4)	0.82	0.65–1.05
Hodgkin lymphoma	210 (2)	120 (3)	0.93	0.67–1.29
Non-Hodgkin lymphoma	1137 (12)	558 (13)	0.96	0.76–1.21
Chronic lymphocytic leukemia	290 (3)	157 (4)	1.04	0.75–1.43
Multiple myeloma	261 (3)	145 (3)	0.86	0.61–1.21
Cancer stage^a^				
I	3030 (31)	1272 (29)	1.0 (ref)	
II	2696 (28)	1138 (26)	0.89*	0.80–0.99
III	1828(19)	669 (15)	1.01	0.88–1.15
IV	738 (7)	355 (8)	0.90	0.77–1.07
Not applicable/unknown	1392 (14)	893 (21)	0.82	0.66–1.01
Initial treatment received				
Surgery	6307 (65)	2534 (59)	1.21	0.99–1.49
Systemic therapy^b^	2780 (29)	1172 (27)	1.10	0.97–1.25
Radiotherapy	2454 (25)	907 (21)	1.18**	1.05–1.32
Hormonal therapy	354 (4)	173 (4)	0.87	0.68–1.12
No therapy/surveillance	633 (7)	377 (9)	0.82*	0.67–0.99
Time between diagnosis and invitation				
< 2 years	2417 (25)	1323 (31)	0.73**	0.64–0.82
2–3 years	1995 (21)	689 (16)	1.00 (ref)	
3–5 years	2234 (23)	905 (21)	0.88*	0.78–0.99
> 5 years	3038 (31)	1410 (33)	0.74**	0.66–0.83
Comorbidities				
0	4352 (45)	1770 (41)	1.0 (ref)	
1	2479 (26)	1045 (24)	0.91	0.83–1.00
≥ 2	1914 (20)	904 (21)	0.84**	0.75–0.93
Unknown	939 (10)	608 (14)	0.67**	0.56–0.79

Significant odds ratios are in bold

*P < 0.01, **P < 0.05

^a^According to TNM. Ann Arbor Code was used for Hodgkin lymphoma and Non-Hodgkin lymphoma. For Chronic lymphocytic leukemia, Multiple myeloma and borderline ovarian tumor stage were not determined or registered

^b^Systemic therapies were chemotherapy, targeted therapy, and immune therapy

**Fig. 1:**
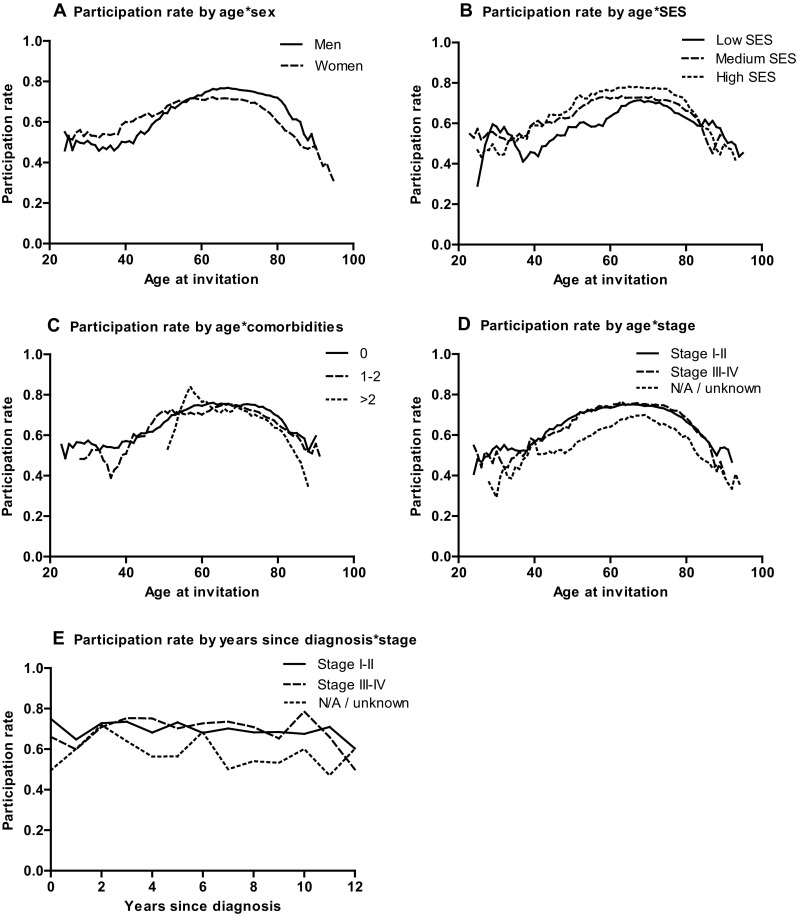
Graphical view of participation rates (participants versus total non-participants) and interacting independent variables. Note: Central moving means of 5 neighboring ages are shown. For years since diagnosis, year averages are shown

In additional analyses, an interaction was found between sex and Hodgkin lymphoma (*P* < .01); women with Hodgkin participated more often than men (OR = 1.47, *P* = .13). Women had higher participation rates at ages < 60 (OR = 1.33, *P* < .01) and lower participation rates at ages > 60 (OR = 0.66, *P* < .01) compared to men. No interactions were found between age and comorbidities, age and stage, age and SES, and time since diagnosis and stage (Fig. 1).

**Fig. 2:**
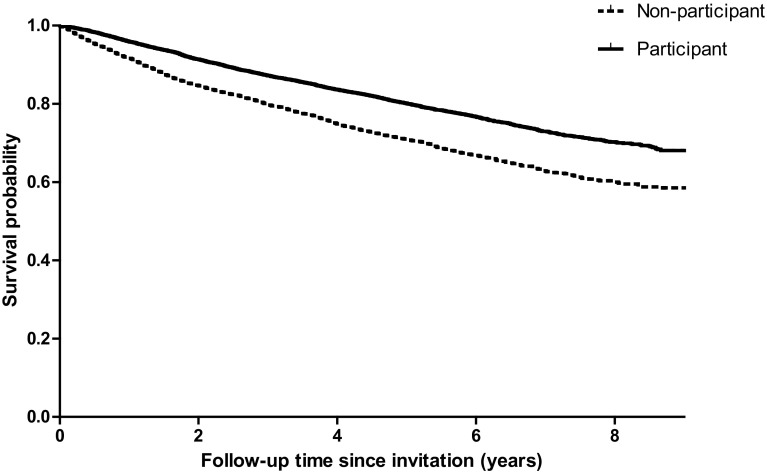
Survival curves of participants versus non-participants, unadjusted

Cox proportional hazard regression models showed that participants had a significantly lower overall survival compared to non-participants (HR = 0.66, *P* < .01), which remained statistically significant after adjustment for covariates (HR = 0.68, *P* < .01) (Table 3; Fig. 2). Results remained similar in survivors with advanced disease (stage 4; HR = 0.75, *P* < .01). Differences in survival between participants and non-participants were not significant in patients younger than 50 (Table 3).

**Table 3:** Risk estimates of participants versus total non-participants on all-cause mortality

	Total, *N*	Deaths, *N*	Person-years	Unadjusted		Adjusted^a^	
				HR (95% CI)	*P* value	HR (95% CI)	*P* value
Total	14,011	3518	68,552.27	0.66 (0.61–0.70)	< **0.01**	0.68 (0.63–0.73)	<.**0.01**
Age groups							
Age < 50	1337	84	7294.05	0.59 (0.73–1.75)	0.59	1.19 (0.74–1.89)	0..47
Age 50–59	1945	296	10357.03	0.77 (0.60–0.98)	**0.03**	0.67 (0.52–0.86)	< **0.01**
Age 60–69	4222	803	21652.54	0.67 (0.58–0.77)	< **0.01**	0.70 (0.60–0.81)	< **0.01**
Age 70–79	4651	1400	22128.11	0.69 (0.61–0.76)	<.**0.01**	0.72 (0.64–0.80)	< **0.01**
Age ≥ 80	1861	935	7120.54	0.58 (0.51–0.66)	<.**0.01**	0.56 (0.49–0.64)	< **0.01**

^a^Analysis was adjusted for age at invitation (continuous), sex, socio-economic status, tumor type, stage, number of comorbidities, primary treatments received (surgery, systemic therapy, radiotherapy, hormonal therapy, no therapy/active surveillance)

The mean estimated HRQoL scores of non-participants matched to participants with similar characteristics were lower than the mean HRQoL of all participants. Mean function scales were estimated to be 1.8–4.3 points lower (worse functioning), and mean symptom scales were estimated to be 0.5–4.8 points higher (more symptoms) among non-participants when compared to participants. In sensitivity analysis, mean function scales of imputed data (participants + matched non-participants) were 0.5–1.3 points lower, and symptom scores were 0.1–0.9 points higher compared to participant data (Table 4).

**Table 4:** Sensitivity analysis comparing health-related quality of life of participant data versus hot deck imputed data

	Participants^a^ *N* = 7368	Participants matched to non-participants^a,b^ *N* = 3348	Imputed data^a,b^ *N* = 10,716	Difference participant data and imputed data
Global quality of life	76.3 (19)	73.0 (21)	75.3 (20)	− 1.0
Physical Functioning	81.1 (19)	78.6 (23)	80.3 (21)	− 0.8
Role Functioning	79.7 (28)	75.6 (32)	78.4 (29)	− 1.3
Emotional functioning	85.0 (20)	83.3 (22)	84.4 (21)	− 0.6
Cognitive functioning	84.6 (21)	83.2 (21)	84.1 (21)	− 0.5
Social functioning	86.5 (22)	84.9 (25)	86.0 (23)	− 0.5
Fatigue	24.1 (25)	28.7 (27)	25.5 (26)	− 1.4
Nausea and vomiting	4.1 (12)	4.4 (14)	4.2 (13)	0.1
Pain	16.7 (25)	18.1 (25)	17.3 (25)	0.6
Dyspnea	14.9 (25)	17.7 (28)	15.8 (26)	0.9
Insomnia	20.6 (29)	23.5 (29)	21.5 (29)	0.9
Appetite loss	6.5 (18)	9.2 (23)	7.3 (20)	0.8
Constipation	9.1 (20)	11.6 (23)	9.9 (21)	0.8
Diarrhea	8.0 (19)	10.0 (23)	8.6 (21)	0.6
Financial difficulties	7.0 (19)	8.0 (20)	7.4 (19)	0.4

^a^HRQoL(EORTC QLQ-C30) was not measured in the earlier study samples (1 and 2, Table 1) and were therefore not included in this sensitivity analysis

^b^Non-participants were matched to participants that completed the HRQoL questionnaire on survival since diagnosis (strata of 2 years), age category (strata of 5 years), sex and tumor type; 3258 out of 3539 non-participants could be matched

## Discussion

Patients who participated in observational PRO research had on average a 32% lower overall survival compared to non-participants over an average period of 9 years after invitation, suggesting a poorer health status among non-participants. This finding is further confirmed by our sensitivity analyses that imply that non-participants may have had HRQoL scores 2–5 points lower than participants, resulting in scores up to 1.3 points lower than initial non-imputed data. Non-participants were on average more often female, aged younger (< 60) or older (> 70), had a lower SES, less often received radiotherapy or no treatment, and had more comorbidities.

Our observed participation rates are similar or even higher compared to other observational PRO studies in cancer patient populations, using similar recruitment strategies [8, 9, 11, 12, 23]. Our average participation rate of 69% is higher than the general rule of thumb of 60%, indicating good quality of research [4]. However, our study shows that participants significantly differ from non-participants on some aspects and may not be fully representative of the population of interest.

The lower survival among non-participants was found for patients aged over 50. In younger patients, no difference was found in survival between participants and non-participants. Non-participation in younger patients may be more often caused by changes in residential address, perhaps even a relatively good HRQoL and not wanting to be reminded of having had cancer [24]. Our findings are in line with a study in a general patient population, which showed that older patients with poorer HRQoL were less likely to participate in survey research, while younger patients with a poorer HRQoL were more likely to participate [10]. To the best of our knowledge, no earlier studies have assessed differences in survival between participants and non-participants of PRO research in any patient population.

Non-participants in the PROFILES registry were more often younger (< 60) or older (> 70). Similarly, ages at both extremes have earlier been associated with lower participation in PRO research among colorectal cancer survivors [8]. However, others did not find any association with age in other cancer types [11, 23]. In cancer clinical trials, young adults as well as the elderly have shown to be largely underrepresented, independent of inclusion criteria [25–27].

Similar to our findings, a lower SES has earlier been associated with lower participation in PRO research among cancer survivors [8, 12, 23]. Further, it was found that women were less likely to participate compared to men. In contrast, in Hodgkin Lymphoma survivors, with an average age of 45, men were less likely to participate than women. Similarly, in a study among non-Hodgkin lymphoma survivors, men had higher participation rates [12], although others did not find any association with sex in other cancer types [8, 9, 11, 23]. Due to our heterogenous sample, it is difficult to establish whether associations with sex were related to psychosocial or etiological differences.

With respect to clinical characteristics, it was found that women with (borderline) ovarian cancer more often did not participate. Also, patients who did not receive any therapy were more often non-participants, whereas patients receiving radiotherapy were more likely to participate. These findings suggest that non-participants may be more often incurable. The most optimal participation rates were observed among patients between 2 and 3 years after cancer diagnosis. At that time patients have completed their treatments, but are still under follow-up. Patients who were invited before 2 years after diagnosis as well as patients invited after 3 years had lower participation rates. Contrary to our findings, other studies among cancer survivors did not find any associations with type of cancer, cancer treatment, or time since diagnosis [9, 11, 23]. This is probably due to the smaller and more homogenous study samples in these studies, limiting detection of substantial differences across clinical variables.

An important strength of the current study is that it included a large and heterogeneous population-based sample of cancer survivors, including both short- and long-term cancer survivors and patients with cancer of 12 different localizations. This allowed us to detect effects of many patient characteristics, and to generalize our results to the larger population of cancer survivors. Another strength is that, through linkage with cancer registry data and the Dutch municipal personal records database, complete and comprehensive data on socio-demographic and clinical characteristics and survival were available for the full population of participants and non-participants. In existing literature, non-participant data are often collected through follow-up surveys or (telephone) screening interviews among initial non-participants. These methods often result in failure to reach all non-participants which may lead to substantial biases, such as an underestimation of differences between participants and non-participants [3, 4].

A limitation of our study is that our study sample is a collection of separate study samples, with different inclusion criteria and sample sizes. However, selections of patients were always based on selection from the NCR and methodology of data collection was similar throughout all studies. This study included a relatively large proportion of colorectal cancer and rare cancer types such as non-Hodgkin lymphoma, while more common cancer types such as breast or lung cancer were lacking. Our study sample may therefore over represent rare cancer types and may not be fully representative of all types of cancer survivors. However, associations with participation were similar across cancer types. Also, some study samples did not include advanced cancer stages, but results were similar in a subgroup analysis including stage 4 only.

Results from our sensitivity analysis demonstrate that hot deck imputation results in lower HRQoL scores, albeit trivial differences on population level [28]. However, imputation and weighting techniques probably still underestimate the HRQoL scores of the population of interest because data are based on participants with a better general health [22, 29]. Therefore, efforts need to be made in patient recruitment to reach those less likely to participate [30]. Recent developments in the integration of PROs in daily clinical practice to monitor patient’s symptoms and HRQoL show high participation rates [31, 32]. Further, providing feedback to patients on their PROs may increase patients’ willingness to participate in PRO research [33].

Our results are of importance as health care decisions are increasingly based on PROs. According to the Patient-Centered Outcomes Research Institute (PCORI), engagement of patients is necessary to make informed health care decisions [34]. However, when PROs are not available from selected patient populations with worse prognosis, we may evaluate effects on PRO outcomes for the relatively healthy subgroup and implement interventions that do not totally fit the population of interest. Even if studies achieve participation rates of around 70% that are generally considered to be fairly good, we may underestimate outcomes. Therefore, strategies to reach those less likely to participate are warranted [30], whereas statistical adjustment techniques should be applied when non-participation bias is prevalent [22, 29].

Due to the relatively high participation rates in population-based observational PRO research, the impact of selection bias is probably smaller when compared to (clinical) trials which mostly have restrictive selection criteria and much lower participation rates. Therefore, we should not disregard population-based research involving PROs which complements clinical trial outcomes in the evaluation of health care interventions.

In conclusion, cancer survivors participating in observational PRO research have a lower survival compared to non-participants, and differ with respect to both socio-demographic and clinical characteristics. Therefore, observational PRO studies may not be fully generalizable to the population of interest and strategies to account for this are warranted.
